# Molecular and clinical characterization of atypical central neurocytomas: implications for diagnosis and treatment strategies

**DOI:** 10.1007/s12672-024-01172-0

**Published:** 2024-07-27

**Authors:** Feixia Sun, Zuocheng Yang, Ronghua Kong, Song Han

**Affiliations:** 1 Shandong Academy of Occupational Health and Occupational Medicine, Shandong First Medical University & Shandong Academy of Medical Sciences Affiliated Occupational Disease Hospital (Shandong Occupational Disease Hospital), Ji’nan, Shandong China; 2 Department of Neurosurgery, Beijing Tiantan Hospital, Capital Medical University, Beijing, China; 3 Shandong Cancer Hospital and Institute, Shandong First Medical University and Shandong Academy of Medical Sciences, Jiyan Road, No. 440, Ji’nan, Shandong China; 4 Department of Neurosurgery, Sanbo Brain Hospital, Capital Medical University, No. 50 Xiang Shan Yi-Ke-Song, Haidian District, Beijing, China

**Keywords:** Atypial central neurocytoma, Central neurocytoma, Classification, Treatment strategies

## Abstract

**Objectives:**

This study aimed to investigate the histological and molecular characteristics of atypical central neurocytomas (CNs) and evaluate their clinical treatment outcomes, with the aim of identifying reliable biomarkers for differentiation and optimal treatment strategies.

**Methods:**

We conducted a retrospective study including 61 patients diagnosed with CNs. Clinical data, neuroimaging, and pathological findings were analyzed. RNA sequencing was performed on tumor tissues to identify differentially expressed genes.

**Results:**

Histological atypia and the Ki-67 index showed no significant impact on progression-free survival (PFS) or overall survival (OS). RNA sequencing identified significant genetic alterations in pathways such as neuroactive ligand–receptor interaction, cAMP, MAPK, and Ras signaling. Differently expressed genes included AMOTL1, PIK3R3, TGFBR1, SMO, COL4A6, MGP, SOX4, IGF2, SLIT1, and CKS2. The five-year OS rate (*p* = 0.015) and PFS rate (*p* = 2.00 × 10^−6^) were significantly higher in the complete resection (CR) group compared to the incomplete resection (IR) group. Postoperative radiotherapy did not affect OS or PFS in the CR group. The five-year PFS rate (*p* = 3.80 × 10^−5^) was significantly longer in patients in the CR group who did not receive radiotherapy compared to those in the IR group who did receive radiotherapy. The extent of surgical resection and operative approaches were found to be irrelevant to perioperative complications and dysfunctions at the last follow-up.

**Conclusion:**

CR is crucial for a better prognosis in patients with atypical CNs. Additional radiotherapy after CR offers little benefit. Histological atypia and the Ki-67 index are not effective in distinguishing between atypical and typical CNs. Identified genetic alterations provide insights into the aggressive behavior of atypical CNs, suggesting potential therapeutic targets and underscoring the need for further research to optimize treatment strategies.

## Introduction

Central neurocytoma (CN) is a rare tumor accounting for 0.1–0.5% of all primary brain tumors. It arises from bipotential precursor cells in the lateral ventricles and/or the third ventricle near the foramen of Monro and is frequently attached to the septum pellucidum [1, 2]. CNs are typically regarded as benign and associated with a favorable prognosis. Nonetheless, some neurocytomas exhibit histological atypia and heightened proliferative activity, leading to adverse clinical outcomes. Atypical CNs demonstrate aggressive behavior, local recurrence, and craniospinal dissemination [2, 3]. Typically, these characteristics are defined by a Ki-67 index greater than 2% and/or histological atypia [4, 5]. However, the prognostic value of both the Ki-67 index and histological atypia remains controversial. The World Health Organization (WHO) classification does not recognize atypical central neurocytomas as a distinct pathological entity [6–9]. To address these challenges and better distinguish between typical and atypical CNs, there is a growing need to leverage advanced genetic analysis techniques, such as RNA sequencing. These methods can provide deeper insights into the molecular differences between typical and atypical CNs, potentially uncovering more reliable biomarkers for differentiation. By exploring the genetic and transcriptomic landscapes of these tumors, researchers hope to identify novel indicators that could more accurately demarcate typical CNs from their atypical counterparts, ultimately leading to improved diagnostic precision and patient outcomes.

Additionally, although there are no standardized guidelines or protocols for the treatment of atypical central neurocytomas, some studies indicate that complete resection (CR) is associated with improved local control rates and overall survival [10, 11]. Aggressive and extensive resection is accompanied by increased complication rates and surgical mortality due to the deep location and proximity to critical structures [12]. Radiotherapy may be beneficial in improving local control and overall survival after incomplete resection (IR), but the side effects, such as mortality due to radiation necrosis, radiation-induced anaplastic astrocytoma, memory impairment, and cognitive functional decline, should not be ignored [5, 13–15]. Therefore, more study are needed to evaluate whether radiotherapy could bring greater benefit to patients or not.

In an effort to address the controversies and gaps in the evaluation system and treatment of atypical CNs, we conducted a retrospective study of their histological and molecular biological characteristics, and analyzed clinical treatment prognosis. Our study aims to contribute to a better understanding of atypical CNs and provide an optimal treatment strategy. By integrating genetic, histological, and clinical data, we hope to offer insights that can lead to more effective and personalized approaches to managing this challenging tumor type.

## Patients and methods

### Clinical data and neuroimaging

We conducted a retrospective single-center study at Beijing Sanbo Hospital of Capital Medical University. A total of 61 patients with CN were enrolled from April 2009 to August 2019, including those who underwent surgery, were diagnosed with CN, and had complete imaging and pathological data, while excluding those with unspecified Ki-67 data, extraventricular neurocytomas, cerebellar or central liponeurocytomas, and an unusual association of ganglioglioma–neurocytoma. We extracted data on the patients’ ages, gender, clinical manifestations, and pathology and imaging findings. The Karnofsky performance score (KPS) of the admitted patients was directly retrieved from the database or newly evaluated according to the detailed description of the cases. During the follow-up period, we evaluated the functional status of the patients, including motor, sensation, memory, and language function.

Based on the operation records, operation videos, and postoperative imaging data, neurosurgery experts and imaging experts divided the degrees of resection into two groups: the complete resection (CR) and IR groups. The extent of tumor removal was evaluated with reference to the criteria made by Jin Wook. Gross total resection (GTR) was classified as CR, while near-total resection (NTR) and subtotal resection (STR) were considered as IR [16].

### Pathological evaluation

The pathological sections were analyzed, reviewed, and recorded by three neuropathologists. The histological features of each neurocytoma were evaluated. Tumors with Ki-67 > 2%, necrosis, vascular proliferation, cell atypia, and mitosis (≥ 3 mitoses/10 high power fields) were classified as atypical central neurocytomas [10]. Patients were provisionally divided into two groups according to the definition: an atypical CN group (50 cases) and a typical CN group (11 cases).

### RNA sequencing and analysis

Two tumor tissues from 50 primary atypical CNs were obtained. Tumor tissues were collected using a multi-point field sampling method, and then divided into two part: one was frozen and stored in liquid nitrogen for RNA extraction, the other was used for analysis by frozen section biopsy and pathological examination.

A total amount of 1 µg RNA per sample was used as input material for the RNA sample preparations. Sequencing libraries were generated using NEBNext^®^ Ultra™ RNA Library Prep Kit for Illumina^®^ (NEB, USA) following manufacturer’s recommendations and index codes were added to attribute sequences to each sample. PCR products were purified (AMPure XP system) and library quality was assessed on the Agilent Bioanalyzer 2100 system.

Raw data (raw reads) of fastq format were firstly processed through in-house perl scripts. In this step, clean data (clean reads) were obtained by removing reads containing adapter, reads containing ploy-N and low-quality reads from raw data. At the same time, Q20, Q30 and GC content the clean data were calculated. All the downstream analyses were based on the clean data with high quality.

Reference genome and gene model annotation files were downloaded from genome website directly. Index of the reference genome was built using Bowtie v2.2.3 and paired-end clean reads were aligned to the reference genome using TopHat v2.0.12 [17]. We selected TopHat as the mapping tool for that TopHat can generate a database of splice junctions based on the gene model annotation file and thus a better mapping result than other non-splice mapping tools [18].

#### Quantification of gene expression level

HTSeq v0.6.1 was used to count the reads numbers mapped to each gene [19]. And then FPKM of each gene was calculated based on the length of the gene and reads count mapped to this gene. FPKM, expected number of Fragments Per Kilobase of transcript sequence per Millions base pairs sequenced, considers the effect of sequencing depth and gene length for the reads count at the same time, and is currently the most commonly used method for estimating gene expression levels.

GTEx Caudate RNAseq reads count data were obtained from GTEx Data Portal database (https://gtexportal.org/home/datasets). Caudate data was selected as control, our central neurocytoma RNA reads count data selected as case. Different expressed genes we performed by DESeq2 (R package), fold change (log2 transformed ) > 1, p value < 0.05 was selected as significant changed threshold [20]. The enrichment of KEGG pathways for differential expressed genes was performed and visualized by clusterProfiler (R package) [21].

The differential expressed genes of another central neurocytoma dataset (GSE35499) was performed by online tools GEO2R (https://www.ncbi.nlm.nih.gov/geo/geo2r/), fold change (log2 transformed) > 1, p value < 0.05 was selected as significant changed threshold. Up-regulated genes in RNAseq and GSE35499 were extracted respectively, overlapping gene list was performed.

### Survival analysis

IBM^®^SPSS ^®^ software, version 25.0 (IBM Corporation, Armonk, NY, USA) was used for the statistical analyses, and GraphPad Prism software, version 7 was used for mapping (GraphPad Software, San Diego, CA, USA). Binary logistic regression analyses were performed. The Kaplan–Meier method was used to evaluate the OS and local tumor control rates. All methods described in this study were performed in accordance with the guidelines and approved by Ethics Committee of Sanbo Brain Hospital (Approval number: SBNK-YJYS-2019-002-01).

## Results

### Patient characteristics

Fifty patients were enrolled, including 28 males and 22 females, with a male-to-female ratio of 1.27:1, the average age of the patients was 29.44 ± 11.08 years old. A majority of patients had symptoms of intracranial hypertension, 38 of them had headache (38/50, 76.00%), 15 of them had nausea and vomiting (15/50, 30.00%), and 10 of them had limb dyskinesia (10/50, 20.00%). The average preoperative KPS score was 78.67 ± 10.96 (ranged from 60 to 100), fifteen patients (15/50, 30.00%) had KPS < 70 (Table 1).

**Table 1:** Clinical characteristics of central neurocytomas patients

Characteristics	Atypical CNs	Typical CNs
	N (%)	N (%)
Patients (n)	50	11
Age, mean years ± SD (range)	29.44 ± 11.08 (17–74)	35.36 ± 9.49 ( 24–59)
Preoperative average ± SD (range)	80.00 ± 9.42( 60–100)	76.00 ± 14.97( 60–100)
Average clinical follow-up duration (range)	39.10 ± 28.90 (1–99) months	30.36 ± 19.83 (3–75) months
Gender		
Male	28 (56.00%)	10 (90.91%)
Female	22 (44.00%)	1 (9.09%)
Symptoms and signs		
Headache	38 (76.00%)	6 (54.55%)
Nausea and vomiting	15 (30.00%)	1 (9.09%)
Limb weakness	10 (20.00%)	2 (18.18%)
Visual deficit	7 (15.00%)	2 (18.18%)
Asymptomatic	5 (10.00%)	2 (18.18%)
Seize	0 (0.00%)	1 (9.09%)
Hearing loss	3 (6.00%)	0 (0.00%)
Memory disturbance	2 (4.00%)	0 (0.00%)
Temporary disturbance of consciousness	1 (2.00%)	0 (0.00%)
Pathological findings		
Ki-67 mean percentage ± SD (range)	7.7% ± 4.31% (1.5-20%)	1.45% ± 0.40% (1-2%)
GFAP positive	11 (22.00%)	2 (18.18%)
Olig-2 positive	2 (10.00%)	1 (9.09%)
P53 positive	27 (54.00%)	3 (27.27%)
EGFR positive	21 (21/43,48.84%)	6 (54.55%)
BCL-2 positive	5 (5/35, 14.29%)	2 (2/10, 20.00%)
PTEN positive	18 (18/25,72.00%)	4 (4/6, 66.67%)
Gross tumor volume mean ± SD	40531.08 ± 40748.00 mm^3^	53807.10 ± 65786.25 mm^3^
Surgical approach		
Supracontal sulcus approach	48 (96.00%)	10 (90.91%)
Corpus callosum approach	2 (4.00%)	1 (9.09%)
Resection extent		
CR (complete resection)	43 (86.00%)	8 (72.73%)
Surgery alone	26 (52.00%)	4 (36.36%)
Surgery + RT	17 (34.00%)	4 (36.36%)
IR (incomplete resection)	7 (14.00%)	3 (27.27%)
Surgery alone	0 (0.00%)	2 (18.18%)
Surgery + RT	7 (14.00%)	1 (9.09%)

a. The final follow-up KPS included the 4 dead patients

### Immunohistochemical study

**Fig. 1:**
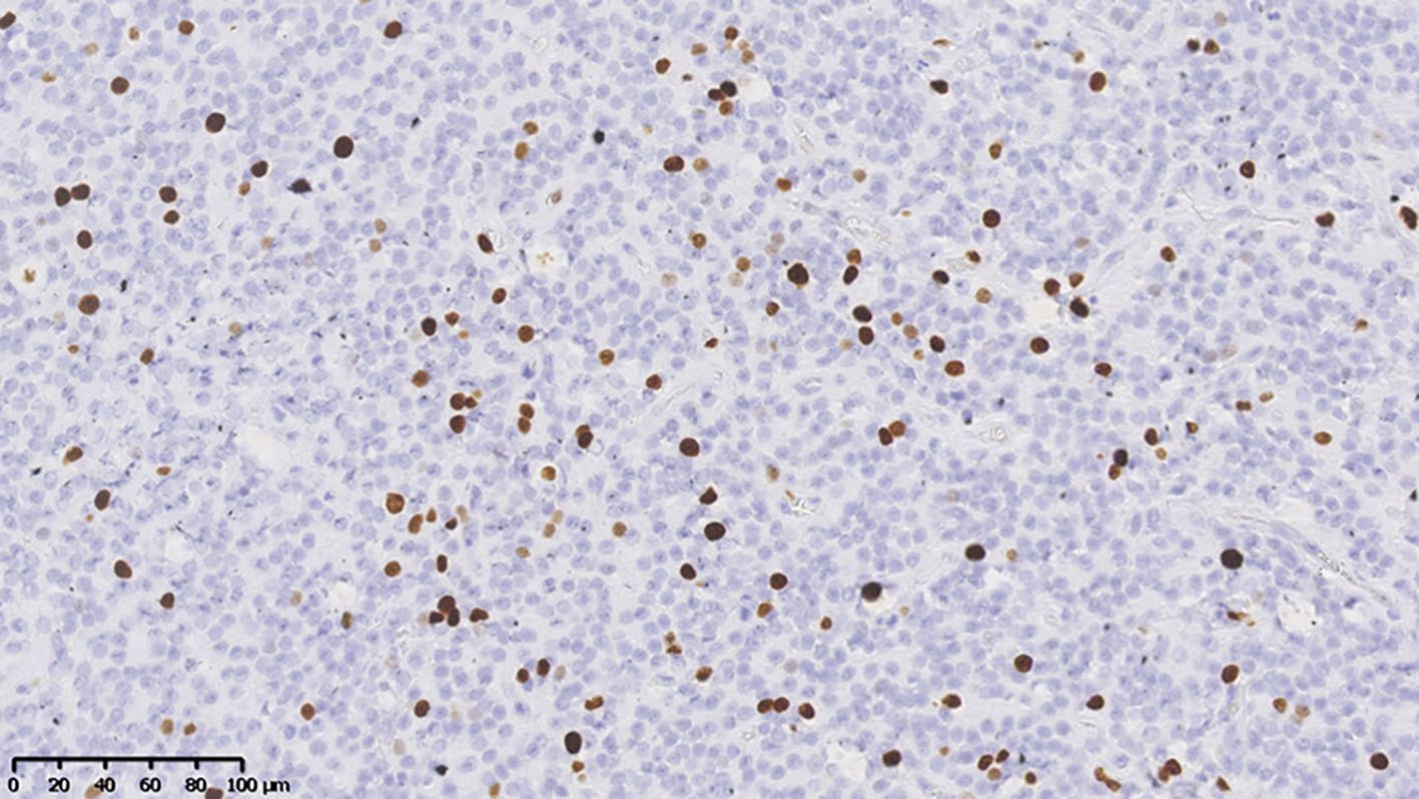
Ki-67 immunostaining shows 15% Ki-67 positive cells

**Fig. 2:**
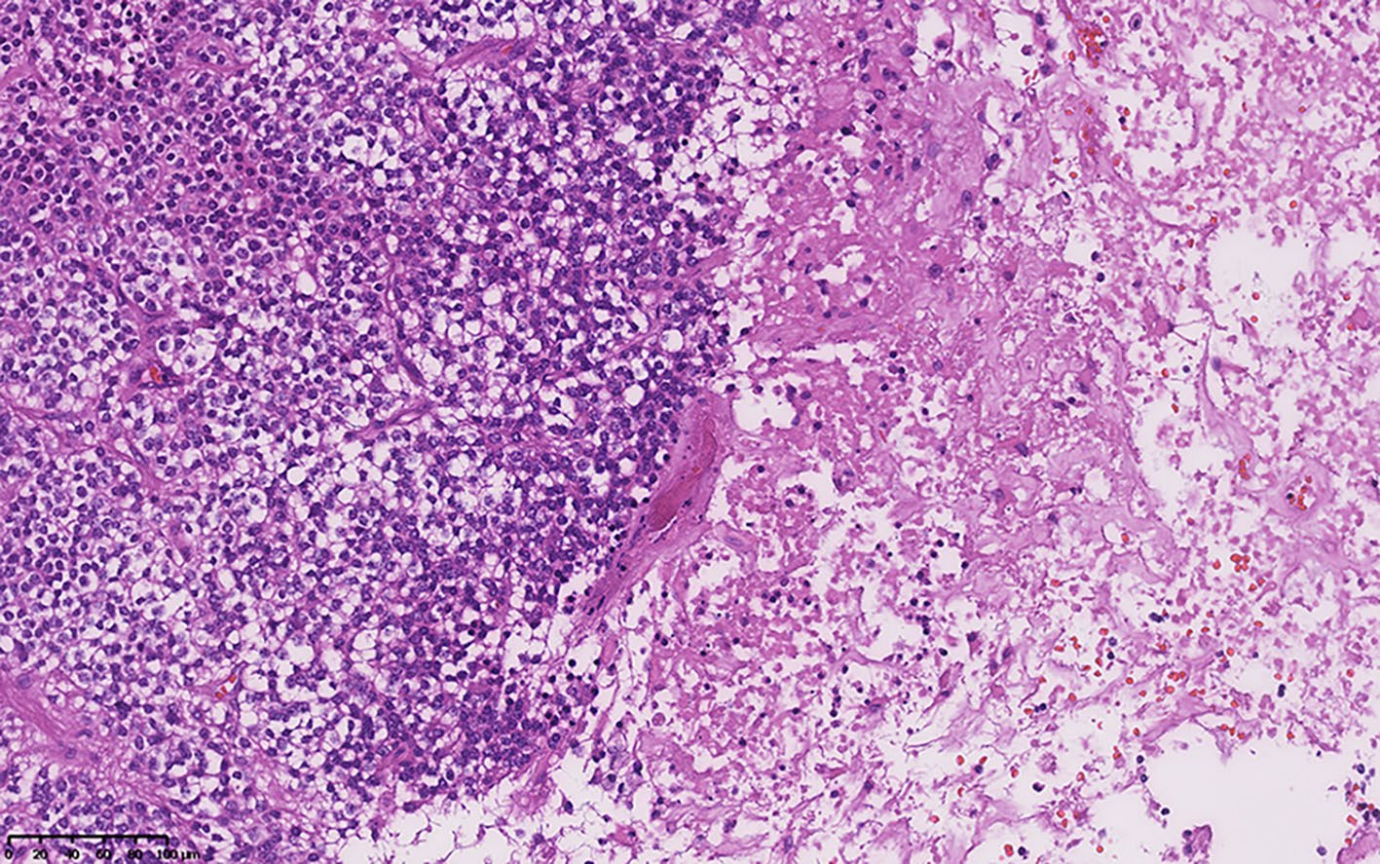
HE staining reveals large necrotic areas

**Fig. 3:**
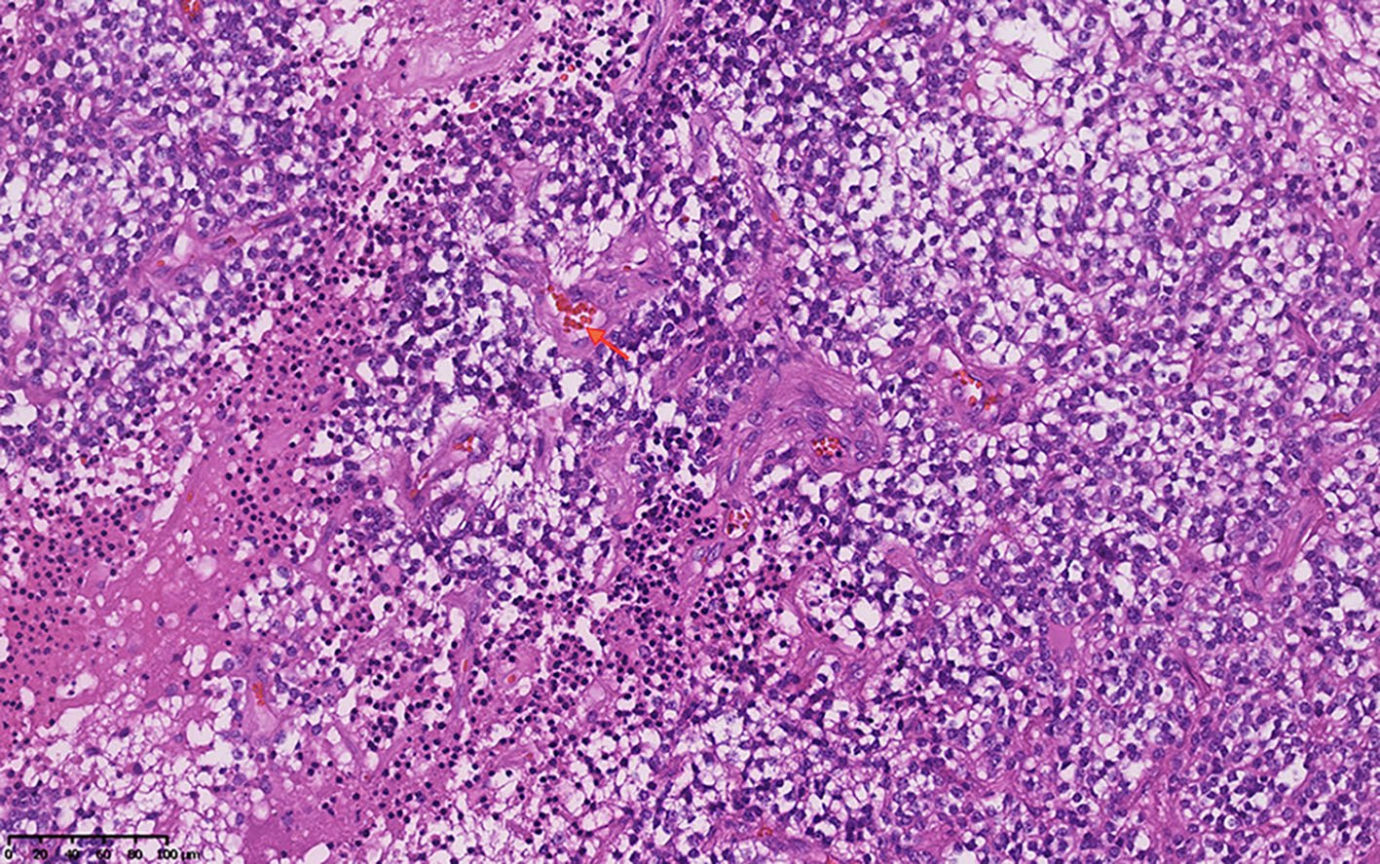
Vascular proliferation (the orange arrow)

The median Ki-67 index was 5.00% in all patients with CNs. The average Ki-67 index was 7.7% ± 4.31% in atypical CNs (Fig. 1), including two cases of vascular proliferation (4.00%, Fig. 2), nine cases of necrosis (18.00%, Fig. 3), one case of nuclear atypia (2.00%), and seven cases of increased mitotic activity (≥ 3 mitoses/10 high power fields) (14.00%). In the atypical CN group, 11 cases (22.00%) were GFAP positive, and 5 cases (10.00%) were olig-2 positive. There were 27 positive P53 cases (54.00%), 21 positive EGFR cases (48.84%), 5 positive BCL-2 cases (14.29%), and 18 positive PTEN cases (72.00%).

### Imaging features

Among this group, 50 cases of atypical CNs were located in the lateral ventricle, and 34 cases (68.00%) of them invaded the third ventricle. The mean volume of atypical CNs was 40531.08 ± 32244.59 mm^3^ (ranging from 2125.11 mm^3^ to 129068.35 mm^3^), with 23 cases (46.00%) exhibiting calcification and 37 cases (74.00%) presenting as cystic solid tumors (Table 1).

### Treatment and survival analysis

**Fig. 4:**
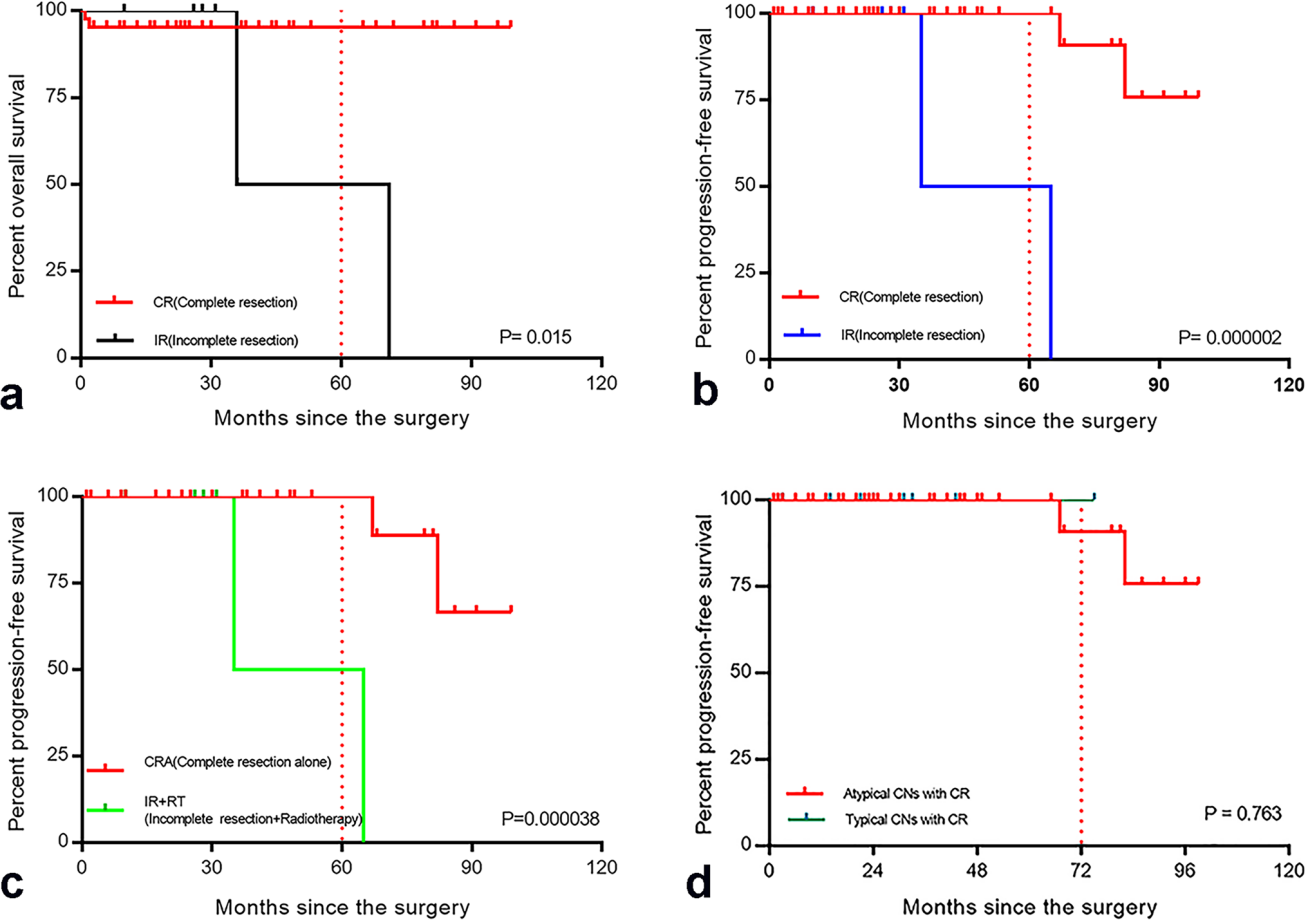
**a** Overall survival stratified by different removal degrees for patients with atypical central neurocytoma (*n* = 50), the 5-year survival rate in the complete resection group was higher than in the incomplete resection group (*P* = 0.015). **b** Progression-free survival stratified by different removal degrees for patients with atypical central neurocytoma (*n* = 50), the 5-year progression free survival rate in the complete resection group was higher than in the incomplete resection group (*P* = 2.00 × 10^−6^). **c **Progression-free survival stratified by varied treatments, the 5-year progression free survival rate among patients in the complete resection group who did not receive radiotherapy was significantly longer than that among patients in the incomplete resection group who received radiotherapy (*P* = 3.80 × 10^−5^). **d** Six-years PFS rate in patients with typical CNs is higher than atypical CNs (100.00%±0.00% vs. 90.91%±8.67%)

In this study, the 5-year and 8-year survival rates were 83.36 ± 8.78%, while the progression-free survival rates were 80.36% ± 10.78% and 69.64% ± 13.67%, respectively (Table 1). In the atypical CNs, the 5-year OS rate (*p* = 0.015, mean ± SD: 95.29 ± 3.25% vs. 50.00 ± 35.35%) and PFS rate (*p* = 2.00 × 10^−6^, mean ± SD: 100% ± 0.00% vs. 50.00 ± 35.35%) were higher in the CR group than in the IR group (Fig. 4). In the complete resection group, postoperative adjuvant radiotherapy had no effect on OS (*p* = 0.255) and PFS (*p* = 0.398). However, the 5-year PFS rate among patients in the CR group who did not receive radiotherapy was significantly longer than that among patients in the IR group who received radiotherapy (*p* = 3.80 × 10^−5^, mean ± SD: 90.00% ± 9.49% vs. 50.00 ± 35.36%) (Fig. 4), and there was no significant effect on OS (*p* = 0.066). The 5-year survival rate (*p* = 4.82 × 10^−4^, mean ± SD: 100% ± 0.00% vs. 65.88% ± 19.96%) and progression-free survival rate (*p* = 0.042, mean ± SD: 100% ± 0.00% vs. 37.50% ± 28.64%) of patients with KPS < 70 were significantly lower than those with KPS ≥ 70.

A multivariate Cox proportional hazards model was used to assess the independent effects of various prognostic factors on PFS and OS in patients with atypical neurocytomas. The results are summarized in Table 2. For PFS, the extent of resection emerged as a significant prognostic factor (B = 2.695, *p* = 0.024, Exp(B) = 14.803), suggesting that complete resection is associated with a markedly reduced risk of progression compared to incomplete resection. Other factors such as gender (*p* = 0.946), age (*p* = 0.495), histological atypia (*p* = 0.636), GFAP positivity (*p* = 0.415), postoperative adjuvant radiotherapy (*p* = 0.121), and preoperative KPS scores (*p* = 0.471) did not show statistical significance as predictors of PFS. Regarding OS, none of the analyzed variables reached statistical significance. The p-values for gender (*p* = 0.350), age (*p* = 0.498), resection extent (*p* = 0.626), histological atypia (*p* = 0.843), GFAP positivity (*p* = 0.887), postoperative adjuvant radiotherapy (*p* = 0.431), and preoperative KPS scores (*p* = 0.632) indicated no significant impact on OS.

**Table 2:** Multivariate Cox analysis of PFS and OS in atypical neurocytoma patients

Variables	Progression-free survival			Overall survival		
	B	*p*-value	Exp(B)	B	*p*-value	Exp(B)
Gender	0.067	0.946	1.070	21.255	0.350	1.70 × 10^9^
Ages	0.027	0.495	1.027	0.483	0.498	1.622
Resection extent	2.695	0.024	14.803	38.262	0.626	4.14 × 10^16^
Histological atypia	0.666	0.636	1.947	3.299	0.843	27.075
GFAP positive	1.122	0.415	3.072	− 2.183	0.887	0.113
Postoperative adjuvant radiotherapy	− 1.942	0.121	0.143	14.933	0.431	3.06 × 10^6^
Preoperative KPS scores	− 0.043	0.471	0.958	0.389	0.632	1.476

*GFAP* glial fibrillary acidic protein, *KPS* karnofsky performance status

In the CR group, the median PFS duration did not differ between the atypical and typical CNs groups (*p* = 0.763) (Fig. 4). The 6-year PFS rate in patients with typical CNs was higher than that in patients with atypical CNs (100.00% ± 0.00% vs. 90.91% ± 8.67%) (Fig. 4). In the IR group, all patients with atypical CNs received postoperative adjuvant radiotherapy, and two patients (28.57%, 2/7) experienced tumor recurrence after surgery. Only one patient received RT after surgery in the three patients with typical CNs, and no one experienced tumor recurrence.

This study analyzed the significance of Ki-67, histological atypia, and GFAP positivity in survival analysis. Although Ki-67 ≤ 5% and > 5% were used to divide typical and atypical CNs groups, no significant difference in PFS was found between the two groups. Based solely on histological atypia, there were no significant differences in PFS (*p* = 0.220) and OS (*p* = 0.341) between the two groups. Immunohistochemical staining for GFAP showed that 13 of the tumor tissues were considered positive (21.31%, 13/61), but it did not affect PFS (*p* = 0.546).

### Function

Postoperative complications mainly included hydrocephalus, limb movement disorder, infection, and bleeding (Table 3). Thirty patients experienced hydrocephalus after surgery, with 9 requiring external drainage and 4 requiring ventriculoperitoneal shunts. In the last follow-up, the KPS score of 35 patients (70.00%) was ≥ 90, and the KPS score of 6 patients (12.00%) was < 70. Although limb movement disorder and long-term memory impairment had no significant correlation with radiotherapy, the proportion of limb movement disorder (20.83% vs. 15.38%) and memory impairment (4.17% vs. 0.00%) at the final follow-up were relatively higher in the postoperative adjuvant radiotherapy group. None of the patients who underwent CR experienced long-term memory impairment, and there was no significant difference in long-term limb movement disorders between patients who received adjuvant radiotherapy and those who did not (*p* = 0.844).

**Table 3:** Postoperative functional outcomes in 50 patients with atypical central neurocytomas

Variable	*N* (%)	*p*-value	
		Surgery approaches	Resection extent
Post-op complications			
Hydrocephalus	25 (50.00%)	0.123	0.820
EVD	7 (12.00%)		
Ventriculoperitoneal shunt	3 (6.00%)		
Motor disturbance	13 (26.00%)	0.470	0.330
Infection	5 (10.00%)	0.618	0.322
Hematoma	4 (8.00%)	0.659	0.382
Aphasia	3 (6.00%)	0.709	0.342
Disturbance of consciousness	1 (2.00%)	0.831	0.672
Visual disturbance	1 (2.00%)	0.833	0.676
The final follow-up average KPS ± SD (range)	85.00 ± 28.16 (0–100)		
Functional outcomes at last follow-up			
Motor disturbance	8 (16.00%)	0.894	0.181
Hydrocephalus	4 (8.00%)	0.670	0.509
Aphasia	2 (4.00%)	0.768	0.560
Seize	1 (2.00%)	0.837	0.684
Hypomnesis	1 (2.00%)	0.999	0.997

*EVD* external ventricular drainage

### Different expressed genes and pathway

**Fig. 5:**
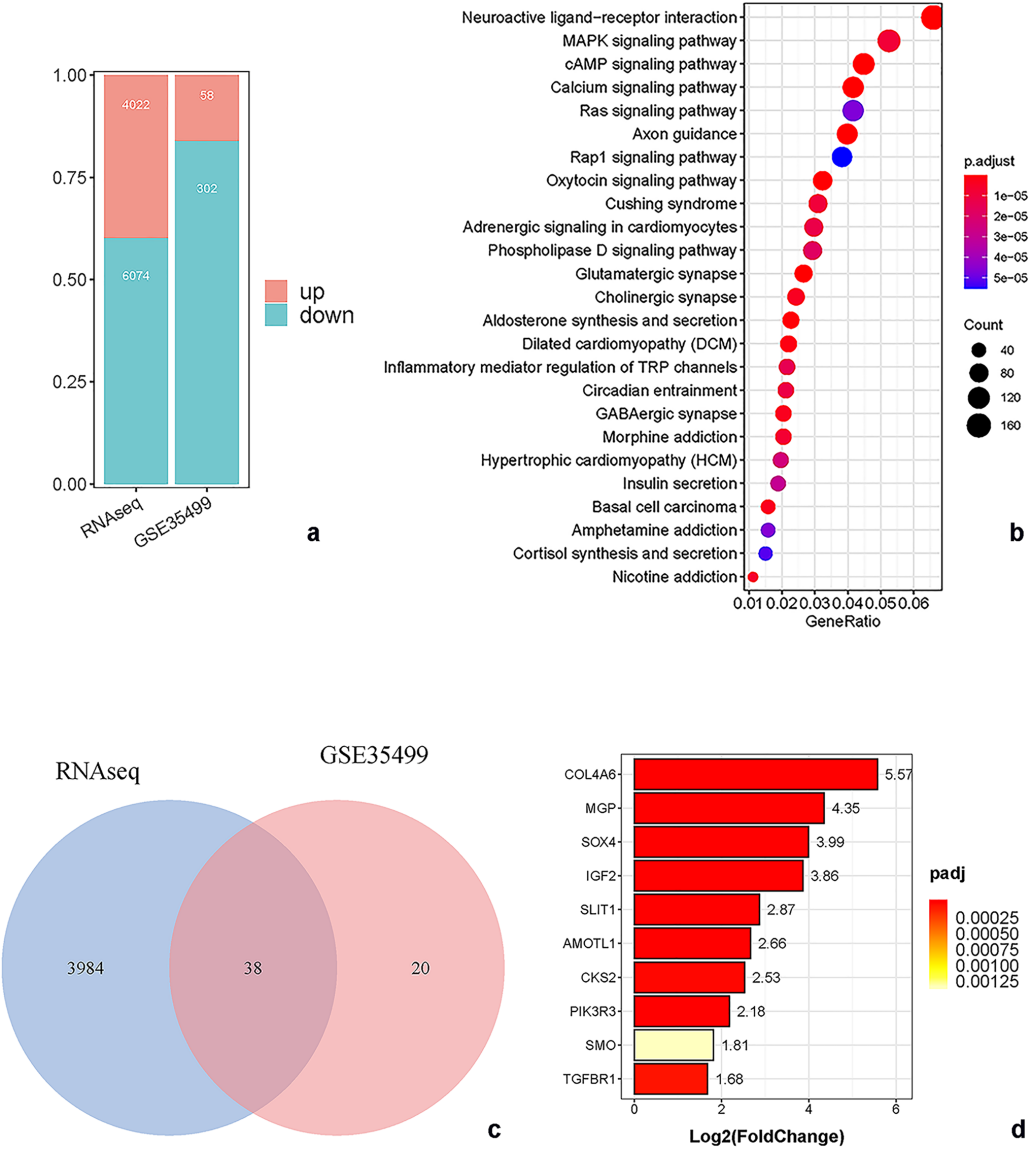
**a **Two datasets result of typical central neurocytoma (RNAseq and GSE35499), differential expressed genes were calculated. RNAseq: 4022 genes up and 6074 genes down. GSE35499: 58 genes up and 302 genes down. **b** Involved pathways of differential expressed genes in RNAseq. Gene numbers and adjusted p value were showed. **c **Overlap genes statistics between RNAseq and GSE35499. Genes up-regulated in both two datasets were selected. 38 genes were obtained finally. (dDisDidssd) Potential malignant related genes, up-regulated (log2 transformed) in RNAseq.

By comparing our samples with caudate control, we got 4022 up-regulated genes and 6074 down-regulated genes (Fig. 5). Further functional analysis, we found these genes involved in some interesting pathways, such as pathways in cancer, Neuroactive ligand-receptor interaction, cAMP, MAPK, Ras, Rap1 pathways (Fig. 5). To narrow down false positive gene candidates, we enrolled another central neurocytoma study (GSE35499), found 58 up-regulated genes and 302 down-regulated genes (Fig. 5). Finally, we selected genes those up-regulated in both studies as our target candidates for further research. We got 38 target genes, in these genes, we found AMOTL1, PIK3R3, TGFBR1, SMO, COL4A6, MGP, SOX4, IGF2, Slit1 and CKS2 genes may contribute to invasion, metastasis and prognosis of central neurocytoma (Fig. 5).

## Discussion

Our study revealed a tendency for tumor recurrence in patients with CNs, even though the current definition of atypical CNs may not have significant prognostic value. Our findings indicate that the current evaluation system may require revision to enhance its predictive accuracy for patient outcomes. Furthermore, CR was significantly associated with improved prognosis, suggesting that radiotherapy may be unnecessary for patients who have undergone complete resection of the tumor.

We found no significant differences in prognosis between atypical and typical CNs, whether classified by the current system or by the median Ki-67 index. Previous studies have used cutoff values of 2%, 3%, and 4% to distinguish between typical and atypical CNs, however, some have suggested that Ki-67 index may be too weak to be a reliable prognostic factor [15, 16, 22]. In our study, the median Ki-67 index was 5.00%, which was higher than that reported in previous studies. This discrepancy may be due to various factors, including potential differences in antibodies, fixatives, and counting methods used across different laboratories. Therefore, Ki-67 may not be suitable as a stable factor to distinguish between typical and atypical CNs. Further studies with larger sample sizes and longer follow-up times are needed to confirm the prognostic value of ki-67 index and the definition of atypical CNs. In addition, we did find a tendency of recurrence in atypical CNs based on the presence of histologically atypical features such as vascular proliferation, necrosis, nucleus atypia, and increased mitotic activity. A multi-center, large sample study indicated that increased mitosis was a single predictor of a high risk of CN recurrence, although this result was not confirmed in a multivariate model [9]. While our study did not find that histologically atypical features had a significant impact on PFS, we observed a tendency towards recurrence in atypical CNs. This finding may underscore the importance of continued monitoring and follow-up in these patients.

Our study’s Kaplan–Meier and Cox multivariate analyses both indicate that CR remains the cornerstone of treatment and is associated with a better prognosis. Postoperative adjuvant radiotherapy may not be necessary for patients who have undergone CR. However, the possibility of tumor recurrence should be closely monitored, even in these patients. Our study revealed that CR can significantly improve PFS and OS in patients with atypical CNs. Previous research has also shown that local radiotherapy can enhance local control in cases of atypical neurocytoma [11]. However, our study found that the addition of radiotherapy to CR had little impact on patient prognosis, as some patients experienced recurrence and death even after CR, highlighting the need for continued vigilance. The use of adjuvant radiotherapy after CR in patients with atypical CNs remains a subject of controversy, and our study suggests that such treatment may not significantly improve patient outcomes. In contrast, Rades and Schild [11]suggested that adjuvant radiotherapy significantly enhances 5-year local control and 5-year survival in cases of atypical neurocytoma treated with partial resection. Nevertheless, the use of radiotherapy after CR in our study did not result in better outcomes compared to those without radiotherapy.

Our study found no significant correlation between surgery-related factors, such as surgical approach and tumor resection degree, and postoperative complications or movement and memory function at the follow-up node. This finding is consistent with the results reported by Lubrano et al. [23], who suggested that gross total resection of CNs did not increase the complication rate compared to subtotal resection of the tumor. Furthermore, Kim et al. [16] found no correlation between different surgical approaches and postoperative complications or KPS score at the last follow-up. Our study’s KPS score at the last follow-up indicated that the patients’ postoperative function had recovered to a greater extent than at the time of visit, providing support for the use of CR as an optimal treatment for atypical CNs. While radiotherapy has been shown to have side effects, including mortality due to radiation necrosis, radiation-induced anaplastic astrocytoma, memory impairment, and cognitive dysfunction [24, 25], our study found no significant correlation between radiotherapy and memory loss at the last follow-up. However, the proportion of patients experiencing memory loss was higher in the postoperative radiotherapy group, which may be attributed to the damage of radiation to the hippocampal structure and corpus callosum.

We found that the proportion of glial marker glial fibrillary acidic protein (GFAP) positivity was slightly higher in the atypical CNs group (22.00%) compared to the typical CNs group. As CNs have features of differentiation along astrocytic and neuronal lineages, immunostaining revealed that neuronal markers synaptophysin (Syn), neuronal nuclear antigen (NeuN), GFAP, and Olig-2 could all be positive simultaneously [5, 26]. Previous research has suggested that positive GFAP may indicate a malignant tendency of the tumor [27, 28]. Our results suggest that GFAP, as a characteristic molecular indicator of glial differentiation, is not sufficient to be used as an indicator for the definition of atypical CNs. In addition, some study showed that CNs lack the 1p/19q codeletion, which could aid in differential diagnosis and potentially impacting treatment strategies [29–31].

Atypical central neurocytomas do not have any known recurrent mutations or chromosomal imbalances, distinguishing them from other neuro-oncological entities [32]. Our study could reveal significant genetic alterations could potentially distinguish between two variants. A previous study found that frequent chromosomal gains of CNs at 2p, 10q, 11q, and 18q, and losses at 1p, 6q, 12q, 17p, 17q, and 20p, with MYCN, PTEN, and OR5BF1 overexpressed and BIN1, SNRPN, and HRAS underexpressed, suggesting MYCN gain and BIN1 loss may contribute to central neurocytoma tumorigenesis [33]. Vasiljevic et al. [34] discovered that certain genes are highly expressed in CNs compared to normal brain tissue, involving the Wnt/β-catenin and Sonic Hedgehog (SHH) signaling pathways. They confirmed the overexpression of CHRDL2, IGF2, KiSS-1, CAL2, NTS, NHLH1, RGS16, and SCGN by real-time RT-PCR. They identified AQP5, KiSS-1, FZD7, AURKB, UBE2C, and PTTG1 as overexpressed in recurrent CNs, suggesting these genes play a role in tumor progression. Among the up-regulated genes AMOTL1, PIK3R3, TGFBR1, SMO, COL4A6, MGP, SOX4, IGF2, Slit1, and CKS2, SMO stands out as a critical component of the SHH signaling pathway. Elevated levels of SMO suggest heightened SHH pathway activity, which is known to drive cell proliferation and sustain stem cell-like properties within tumors [32]. PIK3R3 is part of the PI3K/AKT signaling pathway, which is crucial for cell growth, survival, and metabolism [35]. Among the remaining up-regulated genes, they have been implicated in studies of gliomas or other tumors, where they are associated with cellular processes such as proliferation, differentiation, apoptosis, survival, and even neuronal migration [36–42]. In addition, one study confirmed that methylation analysis cannot distinguish central neurocytoma from atypical central neurocytoma, as both are classified under the “central neurocytoma” methylation class [32]. Another study indicated that methylation profiling on four out of five samples led to modified diagnoses in two patients, indicating that methylation profiling can only help differentiate neurocytomas from similar tumors [43]. Specifically, our RNA detection results differ from previous CNs-related findings, indicating that atypical CNs differentially express genes involved in critical pathways, such as neuroactive ligand-receptor interaction, and the cAMP, MAPK, Ras, Oxytocin, and Rap1 pathways. These pathways are well known for their roles in cell proliferation, survival, and differentiation, suggesting that atypical central neurocytomas may exploit these signaling cascades to drive their aggressive behavior [44–46]. The involvement of these pathways in atypical neurocytomas suggests a complex network of signaling mechanisms that drive the aggressive phenotype of these tumors. Understanding these pathways provides potential targets for therapeutic intervention, aiming to inhibit tumor growth and progression.

The insignificant results obtained in our study may be attributed to various factors, such as inadequate sample size, short follow-up time, and a high proportion of patients treated with CR and/or RT. Notably, the median ki-67 in our patient group was 5.00%, which was higher than that reported in previous studies. The observed difference may be due to variations in antibody, fixative, and counting methods employed in different laboratories [47]. Given the susceptibility of Ki67 to these factors, it may not be a reliable stability factor for the division of atypical CN. Nevertheless, the patients with only histologically atypical features, such as vascular proliferation, necrosis, nucleus atypia, and increased mitotic activity, did not show any significant difference in PFS. In a large-scale multi-center study, increased mitosis was identified as a single predictor of a higher risk of CN recurrence. However, this finding was not confirmed in a multivariate model [9]. Although our results did not show a statistical difference, there was a tendency for recurrence of atypical CNs, as revealed by our findings.

## Conclusion

Our study indicates that CR is crucial for better prognosis in patients with atypical CNs, while additional radiotherapy post-CR could offer minimal benefit. Histological atypia and the Ki-67 index may be not highly effective in distinguishing between atypical and typical CNs from a prognostic perspective. Genetic analysis identified several differentially expressed genes may involve in cancer pathways. These findings provide insights into the molecular basis of atypical CNs and suggest potential therapeutic targets. Further studies are needed to validate these findings and improve treatment strategies for this challenging.

## Data Availability

The clinical datasets generated during and/or analyzed during the current study are available from the corresponding authors on reasonable request.
